# Influence of Cr- and Co-Doped CaO on Adsorption Properties: DFT Study

**DOI:** 10.3390/molecules30132820

**Published:** 2025-06-30

**Authors:** Wei Shi, Renwei Li, Haifeng Yang, Dehao Kong, Qicheng Chen

**Affiliations:** 1 School of New Energy and Materials, Northeast Petroleum University, Daqing 163711, China; 2 College of Mechanical and Electrical Engineering, Jilin Institute of Chemical Technology, Jilin 132022, China; 3 State Key Laboratory of Advanced Welding and Joining, Harbin Institute of Technology, Weihai 264209, China; 4 School of Energy and Power Engineering, Northeast Electric Power University, Jilin 132012, China

**Keywords:** thermochemical energy storage, calcium looping, surface doping, calcium-based composites, DFT calculations

## Abstract

Using the combination of Concentrated solar power (CSP) and calcium looping (CaL) technology is an effective way to solve the problems of intermittent solar energy, but calcium-based materials are prone to sintering due to the densification of the surface structure during high-temperature cycling. In this study, the enhancement mechanism of Co and Cr doping in terms of the adsorption properties of CaO was investigated by Density Functional Theory (DFT) calculations. The results indicate that Co and Cr doping shortens the bond length between metal and oxygen atoms, enhances covalent bonding interactions, and reduces the oxygen vacancy formation energy. Meanwhile, the O^2−^ diffusion energy barrier decreased from 4.606 eV for CaO to 3.648 eV for Co-CaO and 2.854 eV for Cr-CaO, which promoted CO_2_ adsorption kinetics. The CO_2_ adsorption energy was significantly increased in terms of the absolute value, and a partial density of states (PDOS) analysis indicated that doping enhanced the C-O orbital hybridization strength. In addition, Ca_4_O_4_ cluster adsorption calculations indicated that the formation of stronger metal–oxygen bonds on the doped surface effectively inhibited particle migration and sintering. This work reveals the mechanisms of transition metal doping in optimizing the electronic structure of CaO and enhancing CO_2_ adsorption performance and sintering resistance, which provides a theoretical basis for the design of efficient calcium-based sorbents.

## 1. Introduction

The combination of Concentrated solar power (CSP) and calcium looping (CaL) technologies has gained attention as a solution for the problems of intermittent and fluctuating solar energy [1,2]. This integration enables heat to be stored when the sun is out and energy to be released during periods of low or no solar radiation, allowing for precise control and stable power supply even when it is dark [3,4]. CSP-CaL integration has the following advantages: (a) It has a high operating temperature, which enables the implementation of an efficient steam cycle, thus increasing the thermal efficiency of the system [5]. (b) It has a very high energy storage density of about 3.2 GJ/m^3^ [6]. (c) Reactants and products can be safely stored at room temperature without solidification problems [7]. In the CSP-CaL system, the storage and release of thermal energy are accomplished by the calcination and carbonation reaction of the calcium-based material, as shown in the following equations:

(1) $${\mathrm{CaO}}_{\left(s\right)}{+\mathrm{CO}}_{2}\overset{650\;{}^{\circ}C\;<\;T\;<\;850\;\text{°}C}{\to }{\mathrm{CaCO}}_{3\left(s\right)}\;{\Delta H}_{298K}=-178\;\mathrm{kJ}/\mathrm{mol}$$

(2) $${\mathrm{CaCO}}_{3\left(s\right)}\overset{T\;>\;900\;{}^{\circ}C}{\to }{\mathrm{CaO}}_{\left(s\right)}{+\mathrm{CO}}_{2}\;{\Delta H}_{298K}=+178\;\mathrm{kJ}/\mathrm{mol}$$

When the temperature is between 650 °C and 850 °C, CaO reacts with CO_2_ to form CaCO_3_ and release 178 kJ/mol heat. Meanwhile, at temperatures above 900 °C, CaCO_3_ undergoes a calcination reaction to form CaO, and CO_2_ and absorbs 178 kJ/mol heat. However, in a high-temperature and high-CO_2_-concentration carbonation environment, the surface area of calcium-based materials will be significantly reduced, and the porosity will decrease. Meanwhile, a thick layer of CaCO_3_ is rapidly formed at the particle surface, which makes it difficult for CO_2_ to enter the interior of CaO, ultimately leading to a decrease in the effective conversion rate of the material after several cycles [8,9,10].

In response to the problem of the performance degradation of calcium-based materials during high-temperature cycling, researchers have developed a variety of modification strategies, such as thermal activation [11,12,13], mechanical activation [14], controlled reaction pressure [15,16], perturbation [17], and steam activation [18,19]. Notably, the enhancement of calcium-based sorbents through material doping has become an important research direction, and the current doping system types are mainly divided into two categories: The first category consists of alkali metal salt promoters. This type of dopant can effectively promote the reaction kinetics of CO_3_^2−^ generation by enhancing O^2−^ transport efficiency. Meanwhile, these dopants reduce the high-temperature residence time of the sorbent and avoid the structural degradation of the material due to the Taman temperature being exceeded. Huang et al. [20] prepared CaO-based sorbents doped with alkali metal carbonates. The results indicated that the adsorption properties of the sorbents were all superior to those of CaO, and the promotion order was K_2_CO_3_ > Na_2_CO_3_ > Li_2_CO_3_. Al-Mamoori et al. [21] prepared K_2_CO_3_-doped CaO-based sorbents by the precipitation method. The results indicated that K_2_CO_3_ and CaO generate K_2_Ca(CO_3_)_2_ double salts and enhance CO_2_ adsorption properties. The adsorption of CO_2_ by the K-Ca double salt reached the maximum level when the temperature was 650 °C. In our previous work [22], we found that Na_2_SO_4_ doping showed a positive correlation between the effective conversion rate and the doping amount after the first cycle; however, with an increase in the number of cycles and doping concentration, the surface densification of the material intensified, which ultimately led to performance degradation. In addition, for NaCl and KCl doping [23], although alkali metal chlorides significantly improved the reaction kinetics and cyclic stability, the equipment corrosion problems caused by them may affect the safe operating life of the system. For calcium looping technology, maintaining long-term cycling stability is a critical challenge for practical applications. Therefore, another type of dopant is inert oxides. The mechanism of the effect involves inert oxides with excellent thermal stability being uniformly distributed between CaO, which can effectively inhibit the interfacial migration of CaO grains under high-temperature conditions, thus significantly reducing the coarsening rate of the grains. Although a breakthrough has been achieved in experimental studies on the macroscopic scale, further theoretical analysis is still needed on the molecular scale.

Therefore, in this work, we investigated the mechanism of the effect of transition metal Co and Cr doping on the surface structure, electronic properties, and CO_2_ adsorption properties of CaO by DFT calculations. The focus is on the role of doping in oxygen vacancy formation energy, the O^2−^ diffusion energy barrier, CO_2_ adsorption energy, and the particle migration inhibition effect. The mechanism of transition metal doping in optimizing the adsorption performance of calcium-based sorbents is revealed at the atomic scale, which provides theoretical guidance for the design of calcium-based sorbents with high adsorption activity and sintering-resistant properties.

## 2. Results and Discussion

**Figure 1:**
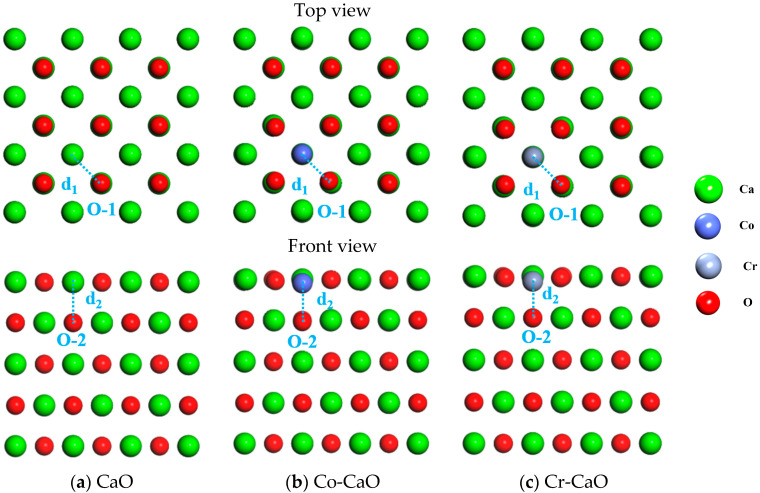
Top and front view plots of CaO, Co-CaO, and Cr-CaO.

In this work, a 2 × 2 × 2 supercell CaO model was constructed with optimized lattice constants of *a* = *b* = *c*= 0.482 nm and *α* = *β* = *γ*= 90°, which is in high agreement with the experimentally determined value of 0.481 nm. Based on the advantages of a small relative change in adsorption energy and a small number of atoms, a five-layer CaO (001) surface model was selected for this study according to the reference [24]. Stable configurations were obtained after the geometrical optimization of CaO, Co-CaO, and Cr-CaO by DFT calculations, and the top and front views are shown in Figure 1. The interatomic distances between the Ca-O, Co-O, and Cr-O atoms are defined as *d*_1_ and *d*_2_, and the values of *d*_1_ and *d*_2_ are shown in Table 1. It can be found that in CaO, the values of *d*_1_ and *d*_2_ are 2.405 Å and 2.397 Å. In Co-CaO, the values of *d*_1_ and *d*_2_ are 2.254 Å and 2.280 Å. In Cr-CaO, the values of *d*_1_ and *d*_2_ are 2.284 Å and 2.124 Å. This indicates that there are interactions between Co atoms and the surrounding O atoms, which consequently shorten the distance of the Co-O bond. Similarly, there are interactions between Cr atoms and the surrounding O atoms, which in consequence shorten the distance of the Cr-O bond.

**Figure 2:**
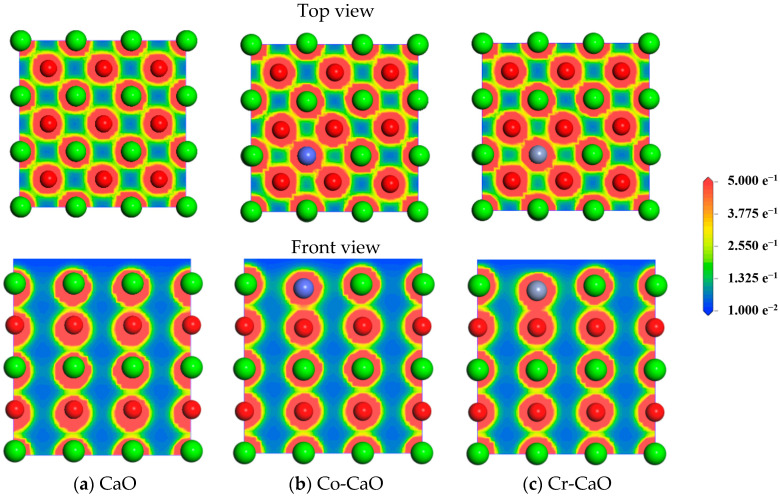
Top-view electron density plot and front-view profile electron density plot for CaO, Co-CaO, and Cr-CaO.

**Figure 3:**
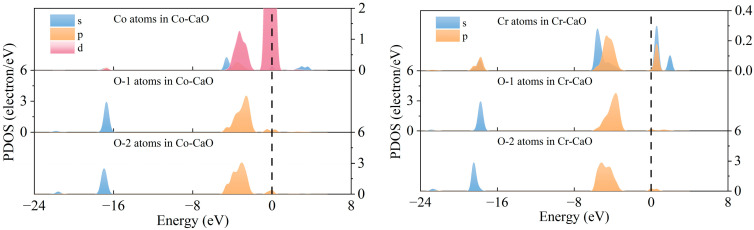
PDOS diagrams of Co atoms and Cr atoms with O atoms.

Figure 2 shows top-view electron density plots and front-view profile electron density plots of CaO, Co-CaO, and Cr-CaO to quantitatively analyze the electronic structures of Co- and Cr-doped CaO surfaces and to reveal the bonding mechanism. It can be found that in Co-CaO and Cr-CaO, the interactions between Co atoms and the surrounding O atoms and Cr atoms and the surrounding O atoms are enhanced due to the overlapping of electrons. Meanwhile, in CaO, the Muliken charge of the Ca atom is 1.294 *e*, the Muliken charge of the O-1 atom is −1.254 *e*, and the Muliken charge of the O-2 atom is −1.226 *e*. In Co-CaO, the Muliken charge of the Co atom is 0.542 *e*, the Muliken charge of the O-1 atom is −1.232 *e*, and the O-2 atom has a Muliken charge of −1.152 *e*. In Cr-CaO, the Muliken charge of the Cr atom is 0.501 *e*, the Muliken charge of the O-1 atom is −1.141 *e*, and the Muliken charge of the O-2 atom is −1.128 *e*. This indicates that Co and Cr doping alters the ionic bond characteristics of CaO by reducing the extent of charge transfer in the central atom and decreasing the electron gain of the O atom, thereby transitioning it toward a covalent bond. In order to show this interaction more intuitively, we performed a partial density of states (PDOS) analysis on Co atoms, O-1 atoms, and O-2 atoms in Co-CaO and Cr atoms, O-1 atoms, and O-2 atoms in Cr-CaO, respectively, as shown in Figure 3. The results indicate that in Co-CaO, there are orbital hybridization peaks between the *d* orbitals of Co atoms and the *p* orbitals in O-1 and O-2 atoms. In Cr-CaO, orbital hybridization peaks also exist between the *p* orbitals of Cr atoms and the *s* and *p* orbitals in O-1 and O-2 atoms. This indicates that in Co-CaO, Co atoms interact with the surrounding O atoms and form Co-O covalent bonds. Similarly, in Cr-CaO, Cr atoms interact with the surrounding O atoms and form Cr-O covalent bonds.

**Figure 4:**
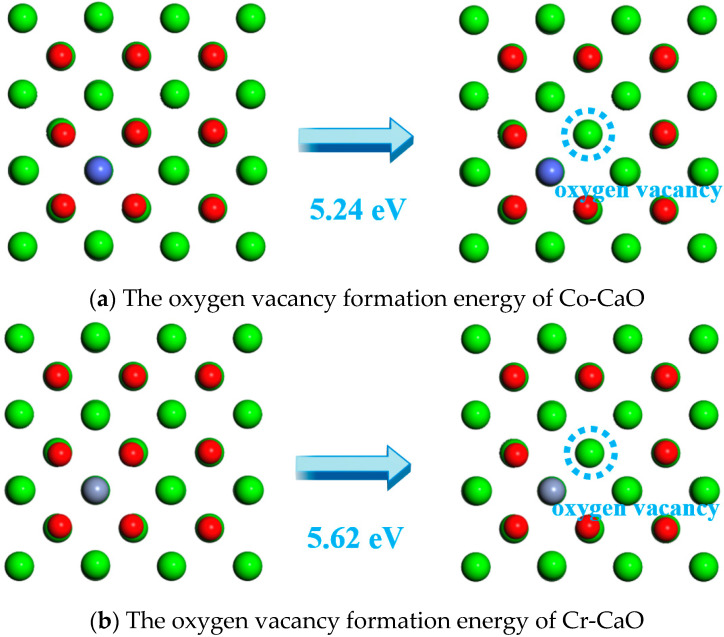
The oxygen vacancy formation energy of Co-CaO and Cr-CaO.

In calcium-based materials, oxygen vacancies enhance the ionic conductivity of the material by forming ion transport channels, which consequently significantly reduces the reaction activation energy. The oxygen vacancy formation energy on the CaO surface was calculated to be 6.84 eV according to Equation (3). Figure 4 shows the oxygen vacancy formation energies on the Co-CaO and Cr-CaO surfaces, which are 5.24 eV and 5.62 eV for the Co-CaO and Cr-CaO surfaces, respectively, and this value is lower than the oxygen vacancy formation energies on the CaO surface. The lower the oxygen vacancy formation energy, the more oxygen vacancies are formed. Therefore, Co and Cr doping makes it easier to form oxygen vacancies on the CaO surface.

**Figure 5:**
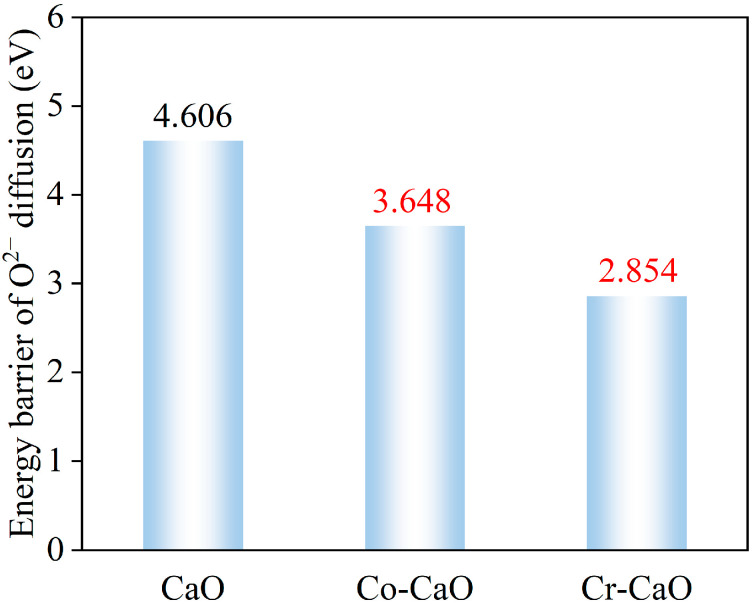
Energy barrier of O^2−^ diffusion for CaO, Co-CaO, and Cr-CaO.

The key step in the carbonation reaction involves the O^2−^ migration process triggered by the formation of surface oxygen vacancies: O^2−^ diffuses from the interior of the CaO lattice to the surface and penetrates the dense product layer to react with external CO_2_ to form CO_3_^2−^. In this process, the diffusion rate of O^2−^ becomes a key step in the carbonation reaction. The energy barrier calculation in Figure 5 shows that the diffusion activation energy of O^2−^ on the CaO surface is 4.606 eV, while on the Co-doped and Cr-doped CaO surfaces, the O^2−^ diffusion activation energy decreases to 3.648 eV and 2.854 eV, respectively. This significant decrease in the energy barrier indicates that the doping of transition metals Co and Cr effectively improves the O^2−^ conductivity of CaO materials. This enhanced O^2−^ diffusion kinetic property directly promotes the chemical binding rate of surface O^2−^ to CO_2_, thus enhancing the carbonation reaction activity of the material.

**Figure 6:**
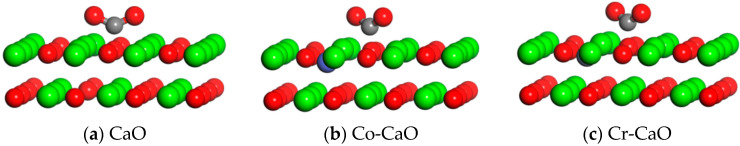
Optimized structure of CO_2_ adsorption by CaO, Co-CaO, and Cr-CaO.

Subsequently, the chemisorption properties of CO_2_ on different surfaces were investigated by placing the optimized CO_2_ molecules at the initial positions of 3 Å on the CaO, Co-CaO, and Cr-CaO surfaces, respectively. The structural optimization results, as shown in Figure 6, indicate that all three material surfaces formed a stable CO_3_^2−^ configuration through the interaction of CO_2_ with surface oxygen atoms, indicating that CO_2_ was chemisorbed by CaO. Table 2 demonstrates the adsorption parameters of CO_2_ on CaO, Co-CaO, and Cr-CaO. On the CaO surface, the bond lengths of CO_2_ are 1.267 Å and 1.267 Å, and the bond angle of CO_2_ is 129.568°, with an *E_ad_* of −1.484 eV. On the Co-CaO surface, the bond lengths of CO_2_ are 1.265 Å and 1.266 Å, and the bond angle of CO_2_ is 127.656°, with an *E_ad_* of −1.659 eV. On the Cr-CaO surface, the bond lengths of CO_2_ are 1.266 Å and 1.267 Å, the bond angle of CO_2_ is 127.836°, and *E_ad_* is −1.587 eV. By comparing the adsorption energy data, it can be found that metal Co and Cr doping significantly improved the CO_2_ adsorption capacity of the materials. This indicating that the transition metal Co and Cr doping effectively improved the CO_2_ capture performance of CaO-based materials, which provides a theoretical basis for the design of efficient CO_2_ sorbents.

**Figure 7:**
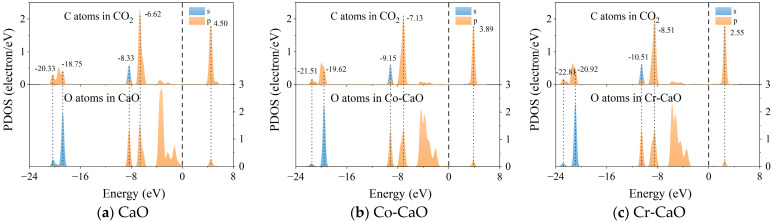
The PDOS diagrams of CO_2_ adsorption by CaO, Co-CaO, and Cr-CaO.

In order to further demonstrate the interaction between the surface O atoms and the C atoms in CO_2_ and to reveal the bonding mechanism, a PDOS analysis of the C and O atoms was carried out, as shown in Figure 7. It was found that there are five obvious resonance peaks between the O atoms on the surface of CaO and the C atoms in CO_2_, which are −20.33 eV, −18.75 eV, −8.33 eV, −6.62 eV, and 4.50 eV, indicating that there is a strong orbital hybridization between the O atoms and the C atoms and the formation of a stable chemical bond. A similar situation also occurs on the surface of Co-doped and Cr-doped CaO. There are obvious resonance peaks at −21.51 eV, −19.62 eV, −9.15 eV, −7.13 eV, and 3.89 eV between O atoms on the surface of the Co-CaO and C atoms in CO_2_, and there are obvious resonance peaks at −22.81 eV, −20.92 eV, −10.51 eV, −8.51 eV, and 2.55 eV between O atoms on the surface of Cr-CaO and the C atoms in CO_2_. It is noteworthy that the characteristic peaks on the surface of Co-doped and Cr-doped CaO both show downward shifts in energy, indicating that the introduction of the transition metals Co and Cr significantly enhances the hybridization strength of the C-O orbitals. This change in electronic structure is highly consistent with the trend in adsorption energy change, i.e., stronger orbital interactions lead to elevated CO_2_ adsorption energy, which reveals the intrinsic reason for the elevated CO_2_ trapping ability of Co and Cr doping at the electronic level.

**Figure 8:**
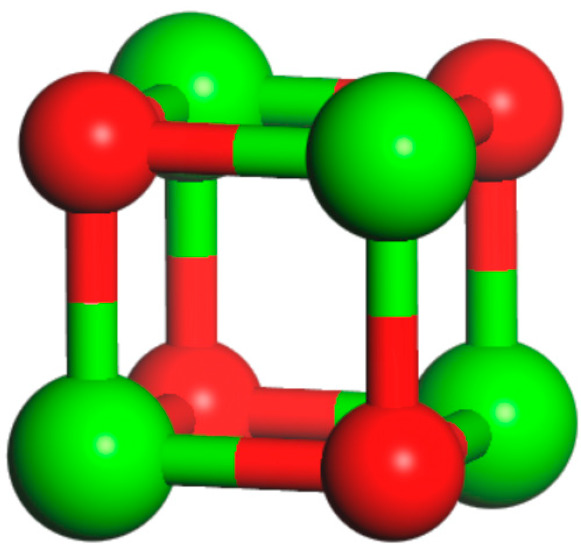
Structure of Ca_4_O_4_.

It was demonstrated by experimental methods that inert oxide doping CaO can form inert carriers with high Taman temperatures, which will be uniformly distributed in CaO, and their role is to prevent the agglomeration of CaO particles by means of physical segregation. Meanwhile, the sintering process involves interactions between nanoparticles, which constantly move and eventually form larger clusters. Therefore, a Ca_4_O_4_ model was first constructed, after which the model was placed onto CaO, Co-CaO, and Cr-CaO surfaces to investigate the interactions between the grains. The Ca_4_O_4_ model was built based on the CaO model, and in order to eliminate the intermolecular interactions, the Ca_4_O_4_ model was placed into a 10 Å × 10 Å × 10 Å box for geometry optimization. The final optimized model diagram is obtained, as shown in Figure 8, and the energy of the optimized model is recorded.

**Figure 9:**
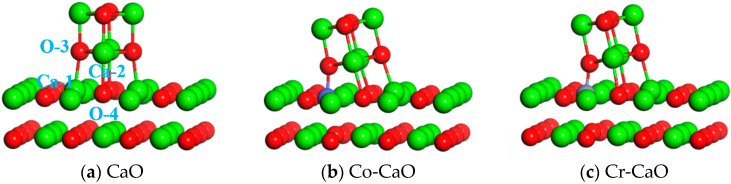
Optimized structural of Ca_4_O_4_ adsorption on CaO, Co-CaO, and Cr-CaO.

The optimized Ca_4_O_4_ cluster models were placed at 3 Å above the CaO, Co-CaO, and Cr-CaO surfaces for structural optimization, and their stable configurations are shown in Figure 9. Calculations indicate that Ca-O bonds are formed between the Ca and O atoms on the surface and the O and Ca atoms in Ca_4_O_4_ in the CaO surface. On the Co-CaO surface, Co-O and Ca-O bonds are formed between the Co and O atoms on the surface and the O and Ca atoms in Ca_4_O_4_. On the Cr-CaO surface, Cr-O bonds and Ca-O bonds are formed between the Cr and O atoms on the surface and the O and Ca atoms in Ca_4_O_4_. Table 3 demonstrates the bond lengths as well as adsorption energy data for the chemical bonds of Ca_4_O_4_ after adsorption on the CaO, Co-CaO, and Cr-CaO surfaces. The results indicated that the adsorption energy of Ca_4_O_4_ adsorbed on the surface of CaO is −2.84 eV, and the length of Ca-1-O-3 after stabilization is 2.32 Å, and the length of Ca-2-O-4 is 2.38 Å. The adsorption energy of Ca_4_O_4_ adsorbed on the surface of Co-CaO is −3.02 eV, and the length of Co-O-3 after stabilization is 1.93 Å, and the length of Ca-2-O-4 is 2.27 Å. The adsorption energy of Ca_4_O_4_ adsorbed on the surface of Cr-CaO is −3.36 eV, and the length of Cr-O-3 after stabilization is 1.88 Å, and the length of Ca-2-O-4 is 2.31 Å. This result indicates that the interaction between Ca_4_O_4_ and Co-doped and Cr-doped CaO surfaces is stronger, and Ca_4_O_4_ clusters can be better adsorbed on Co- and Cr-doped CaO surfaces. Therefore, Co and Cr doping can well relieve the sorbent sintering problem caused by the migration of Ca_4_O_4_ clusters.

## 3. Simulation Details

In this work, the Cambridge Sequential Total Energy Package (CASTEP) computational module based on Density Functional Theory (DFT) was used to investigate the enhancement mechanism of Co and Cr doping on the adsorption properties of CaO [25]. The computational procedure employs the Perdew–Burke–Enzerh (PBE) exchange correlation generalization under Generalized Gradient Approximation (GGA) [26,27,28]. The PBE functional was selected for this study as it has been extensively validated and widely employed for calculating the structural and electronic properties of metal oxides, including CaO-based systems, providing reliable results comparable to experimental observations, facilitating a direct comparison of the results. Meanwhile, we tested the cutoff energy and k-point, as shown in Figure S1. Finally, the cutoff energy was set to 720 eV, and the k-point was set to 2 × 2 × 2 to ensure the convergence of the Brillouin zone integral. To obtain a reliable structure, the system optimization process sets strict convergence criteria: the energy convergence criterion is less than 10^−5^ eV, the maximum force is less than 0.03 eV/Å, the maximum stress is less than 0.05 GPa, and the maximum displacement is less than 10^−3^ Å.

Equation (3) defines the oxygen vacancy formation energy:

(3) $$E_{f}^{1/2O_{2}}=E(reduced)+E(1/2O_{2})-E(stoichiometric)$$

where *E*(*reduced*) is the energy of the surface containing O vacancies, *E*(1/2*O*_2_) is the energy of the O atom in O_2_, and *E*(*stoichiometric*) is the energy of the perfect surface. A lower energy indicates that O vacancies are more likely to form. The linear synchronous transit (LST) and quadratic synchronous transit (QST) methods [29,30] are used to calculate the diffusion activation energy of O^2−^, as shown in Equation (4).

(4) $$E_{a}=E_{TS}-E_{IS}$$

where *E_a_* is the activation energy of the reaction, *E_TS_* is the energy of the diffusion transition state, and *E_IS_* is the energy of the diffusion initial state. A smaller *E_a_* indicates that the O atoms dissociated from the lattice diffuse more easily from the interior to the surface. The adsorption energy (*E_ad_*) is used to express the strength of the adsorption properties of the sorbent, as shown in Equation (5).

(5) $$E_{ad}=E_{CO_{2}+surface}-E_{CO_{2}}-E_{surface}$$

where *E_CO_*_2*+surface*_ denotes the total energy of CO_2_ and the surface, and *E_CO_*_2_ and *E_surface_* denote the energy of CO_2_ alone and the energy of the surface, respectively. The greater the absolute value of *E_ad_*, the greater the ability of the surface to adsorb CO_2_. In addition, the partial density of states (PDOS) of the atoms is obtained using the OptaDOS program, and the Muliken charge is analyzed to evaluate the charge transfer between the atoms [31,32].

## 4. Conclusions

In this paper, the mechanism of the effect of Co and Cr doping on the adsorption performance of CO_2_ on CaO was investigated by DFT calculations, which revealed the effect of doping on the structure, adsorption performance, and sintering resistance of the material, and the main conclusions are as follows:

(a) Co and Cr doping significantly shortens the bond lengths to 2.254 Å for Co-O in Co-CaO and 2.124 Å for Cr-O in Cr-CaO, indicating that the dopant atoms form stronger covalent bond interactions with the surrounding O atoms. This structural change enhances the stability of the material and promotes the formation of surface-active sites. Meanwhile, Co and Cr doping reduces the oxygen vacancy formation energy on the CaO surface, which indicates that it is easier for the doped material to form oxygen vacancies. In addition, the O^2−^ diffusion energy barrier was significantly reduced, indicating that doping increased the O^2−^ migration rate, which accelerated the CO_2_ chemisorption process.

(b) The absolute value of the CO_2_ adsorption energy of the doped materials increased, indicating that Co and Cr doping enhanced CO_2_’s ability to bind to the surface. It was further confirmed by PDOS analysis that the orbital hybridization of dopant atoms with O atoms enhanced the C-O bonding interactions, which provided a theoretical basis for adsorption performance enhancement at the electronic level.

(c) The Ca_4_O_4_ cluster adsorption calculations revealed the formation of M-O bonds between the dopant atoms and clusters and higher adsorption energies between the doped Co-CaO and Cr-CaO surfaces and clusters. This indicates that Co and Cr doping can effectively inhibit the migration and sintering of CaO particles by enhancing surface bonding, thus enhancing the cyclic stability of the materials.

In conclusion, Co and Cr doping significantly enhanced CO_2_ adsorption performance and sintering resistance by optimizing the electronic structure of CaO and reducing the oxygen vacancy formation energy and diffusion energy barrier. This work provides theoretical guidance for the design of efficient and stable calcium-based CO_2_ sorbents and establishes an important foundation for subsequent experimental studies and practical applications.

## Figures and Tables

**Table 1:** Changes in bond lengths between atoms.

Structure	*d*_1_ (Å)	*d*_2_ (Å)
CaO	2.405	2.397
Co-CaO	2.254	2.280
Cr-CaO	2.284	2.124

**Table 2:** Adsorption parameters for CO_2_ adsorption on CaO, Co-CaO, and Cr-CaO.

Structure	Bond Length of CO_2_ (Å)	Bond Angle of CO_2_ (°)	*E_ad_* (eV)
CaO	1.267/1.267	129.568	−1.484
Co-CaO	1.265/1.266	127.656	−1.659
Cr-CaO	1.266/1.267	127.836	−1.587

**Table 3:** Adsorption parameters for Ca_4_O_4_ adsorption on CaO, Co-CaO, and Cr-CaO.

Structure	Length of Ca-1-O-3 or M-O-3 (Å)	Length of Ca-2-O-4 (Å)	*E_ad_* (eV)
CaO	2.32	2.38	−2.84
Co-CaO	1.93	2.27	−3.02
Cr-CaO	1.88	2.31	−3.36

## Data Availability

The original contributions presented in this study are included in the article. Further inquiries can be directed to the corresponding author.

## Supplementary Materials

The following supporting information can be downloaded at https://www.mdpi.com/article/10.3390/molecules30132820/s1, Figure S1: Convergence judgment of CaO.
