# Supplementary material for: Influence of Cr- and Co-Doped CaO on Adsorption Properties: DFT Study
Source: Molecules. 2025 Jun 30;30(13):2820. doi: 10.3390/molecules30132820 (PMC12250958; doi:10.3390/molecules30132820)
Supplement: Supplementary file 1 [file molecules-30-02820-s001.zip › molecules-3704292-supplementary.pdf]

Supplementary Material

Influence of Cr- and Co-Doped CaO on Adsorption Properties: DFT Study

Wei Shi [1], Renwei Li [2], Haifeng Yang [3], Dehao Kong [1,*] and Qicheng Chen [4]

[1] School of New Energy and Materials, Northeast Petroleum University, Daqing 163711, China
[2] College of Mechanical and Electrical Engineering, Jilin Institute of Chemical Technology, Jilin 132022, China
[3] State Key Laboratory of Advanced Welding and Joining, Harbin Institute of Technology, Weihai 264209, China
[4] School of Energy and Power Engineering, Northeast Electric Power University, Jilin 132012, China
* Correspondence: kongdehao1997@163.com

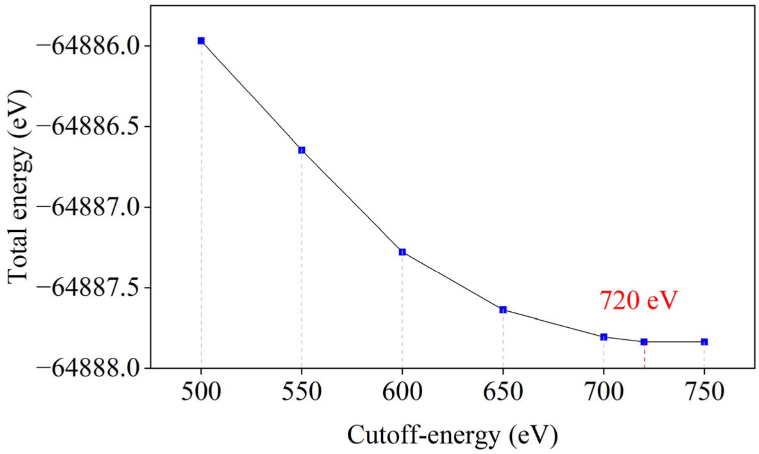

(a) Convergence relationship between cut-off energy and total energy.

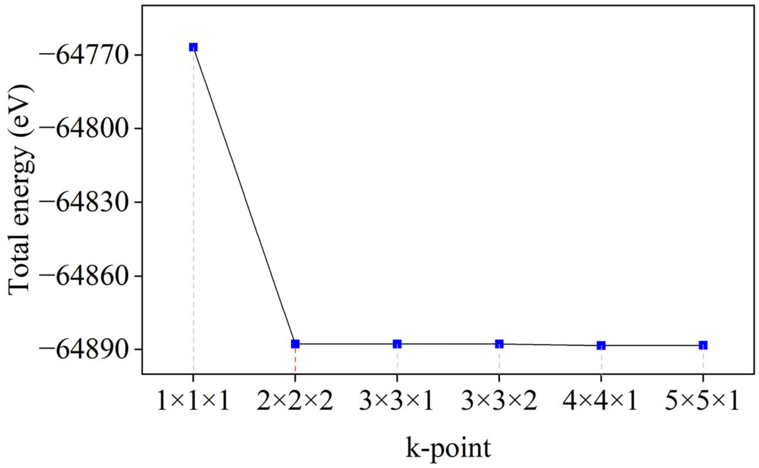

(b) Convergence relationships between k-point and total energy.

Figure S1. Convergence judgment of CaO.
